# An Ultra-High Performance Liquid Chromatographic-Tandem Mass Spectrometric Method for the Determination of Sinomenine in Human Plasma after Transdermal Delivery of the Zhengqing Fengtongning Injection

**DOI:** 10.3390/molecules20046454

**Published:** 2015-04-10

**Authors:** Tingbo Chen, Zheng Xiang, Gengting Dong, Pei Luo, Ping Qiu, Shenzhi Wang, Baoming Huang, Yingyi Wen, Feichi Wu, Liang Liu, Hua Zhou

**Affiliations:** 1 State Key Laboratory of Quality Research in Chinese Medicine, Macau University of Science and Technology, Avenida Wai Long, Taipa, Macau, China; E-Mails: ctb1257@163.com (T.C.); zxiang@must.edu.mo (Z.X.); donggengting@163.com (G.D.); pluo@must.edu.mo (P.L.); 365oa4z2gbc@163.com (B.H.); 2 Hunan Zhengqing Pharmaceutical Group Limited, Huaihua 418005, China; E-Mails: qiu_zq@163.com (P.Q.); wenhui7519@163.com (Y.W.); feichiwu_zq@163.com (F.W.); 3 The First Hospital of Hunan University of Chinese Medicine, Changsha 410007, China; E-Mail: wangshenzhi2006@126.com

**Keywords:** sinomenine, Zhengqing Fengtongning Injection, transdermal delivery, human plasma, concentration, liquid chromatography-tandem mass spectrometry

## Abstract

A sensitive, precise and selective ultra-high performance liquid chromatography method coupled with triple-quadrupole mass spectrometry was developed and validated for the determination of trace amounts of sinomenine (ng/mL) in minute volumes of human plasma. Fifty microliter plasma samples were precipitated using methanol to extract sinomenine. Separation was carried out on a C_18_ column with a water and acetonitrile mobile phase gradient with formic acid as an additive. The mass spectrometry data were obtained in the positive ion mode, and the transition of multiple reactions was monitored at *m/z* 330.2→181.0 for sinomenine quantification. The working assay range for sinomenine was linear from 0.1173 to 15.02 ng/mL with the lower limit of quantification of 0.1173 ng/mL. The precision and accuracy of the method was less than 15% in intra-day and inter-day experiments with a matrix effect of less than 6.5%. After validation, the quantitative method was applied to analyze sinomenine levels in human plasma after transdermal delivery of the Zhengqing Fengtongning Injection. The results showed that some samples contained sinomenine within the concentration range 0.4131–4.407 ng/mL.

## 1. Introduction

**Figure 1:**
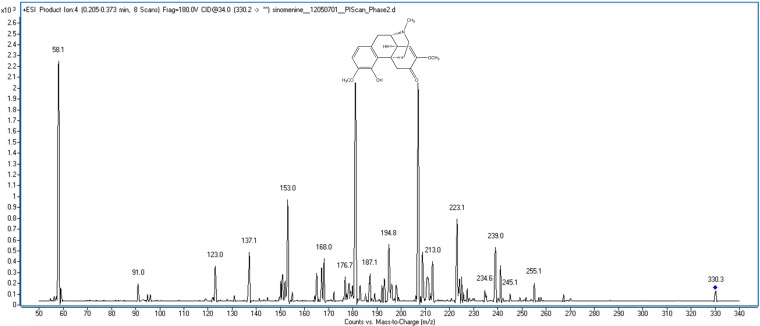
The chemical structure and mass spectra of sinomenine.

Sinomenine (7,8-didehydro-4-hydroxy-3,7-dimethoxy-17-methyl-9α, 13α, 14α-morphinan-6-one, Figure 1) is the main active ingredient from a Chinese medicinal plant *Sinomenium acutum* [1], and has been developed into a series of Chinese proprietary medicines called Zhengqing Fengtongning (FTN) for treating rheumatoid arthritis (RA) and other autoimmune diseases in China due to its anti-inflammatory [2,3] and immunosuppressive actions [4].

Injections and tablets are two kinds of FTN dosage forms. FTN is systemically administrated with low plasma concentrations at lesion sites due to the lack of blood vessels in the joints. Adverse drug reactions occur occasionally with frequent FTN usage, especially when administered via injection [5,6]. Therefore, it is necessary to develop a new administration route for sinomenine. Topical drug delivery systems allow the drug to accumulate in the lesion sites with reduced systemic exposure and may therefore reduce the systemic adverse reactions. In a pilot clinical study, FTN injections transdermal electroporation to the skin areas localized near the lesions was carried out and achieved better therapeutic effects (Wang, S.Z.; Qiu, P. Clinical efficacy of Zhengqing Fengtongning injection transdermal electroporation on rheumatoid arthritis patients. The First Hospital of Hunan University of Chinese Medicine, Changsha; Hunan Zhengqing Pharmaceutical Group Limited, Huaihua, China. Unpublished work, 2015). However, detailed information regarding pharmacokinetics of the transdermal administration in humans remains lacking.

Sinomenine levels in biological samples are normally quantified using high performance liquid chromatography with an ultraviolet detector (HPLC-UV) [7]. However, the *in vivo* test of sinomenine transdermal administration showed a lower C_max_ and area under the concentration-time curve (AUC) in patch and spray formulations compared with an oral preparation [8]. In the previous studies, analytical methods have been developed for sinomenine quantification in body fluids by HPLC with an ultraviolet detector (UV) [9,10,11,12], which had a LLOQ of approximately 0.1 μg/mL [11] and was unsuitable for the quantification of trace sinomenine amounts at the ng/mL level. Sensitive methods for detecting the concentration of sinomenine in human [13,14] or rat [11] plasma with liquid chromatography and tandem mass spectrometry (LC-MS/MS) have also been developed, and had LLOQs of 0.5 ng/mL and 10 ng/g, respectively. However, these methods required a large amount of test samples, and approximately 200 μL plasma [13,14]. Therefore, the aim of this study was to develop a sensitive method for the determination of trace amounts of sinomenine with minute plasma samples volumes. In this paper, a more sensitive analytical method that uses ultra high performance liquid chromatography coupled with tandem mass spectrometry (UHPLC-MS/MS) was developed and validated for the quantification of sinomenine in human plasma. The applicability of using this method to detect sinomenine in humans following transdermal administration is also described.

## 2. Results and Discussion

### 2.1. UHPLC-MS/MS Method Development

**Figure 2:**
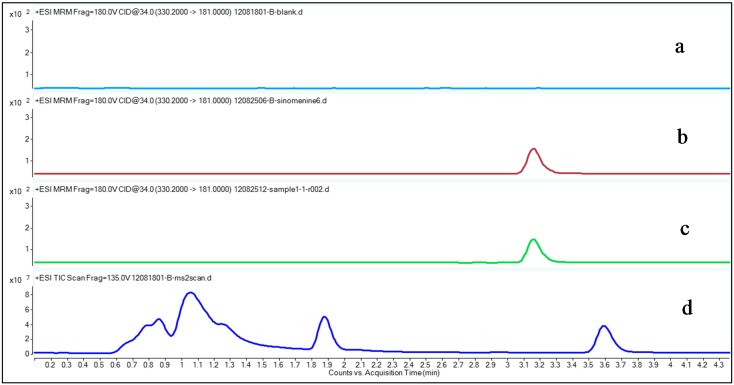
Typical multiple reaction-monitoring (MRM) chromatograms of sinomenine and MS2 scan chromatogram of drug-free plasma. (a) Drug-free plasma; (b) Pre-spiked sinomenine in the drug-free control plasma with a final concentration of 1.878 ng/mL; (c) Sample from a rheumatoid arthritis (RA) patient 30 min after transdermal delivery of the Fengtongning (FTN) injection; (d) MS2 scan result of drug-free plasma.

To optimize chromatographic conditions for determining sinomenine levels from plasma samples by UHPLC-MS/MS, the mobile phase and gradient elution were intensively investigated. Due to the resolution, retention time, and sinomenine sensitivity, formic acid in water was chosen for the mobile phase and acetonitrile was chosen for the gradient elution; this was described in Section 3.2. The formic acid was added to make sure that sinomenine was measured in its salt form. The gradient elution began with a low ratio of acetonitrile to 0.1% formic acid to keep the retention time of sinomenine different from the interference peaks of intrinsic substances, which is shown in the MS2 scan result (Figure 2d).

Sinomenine is an alkaloid, which can easy accept one [H]^+^, and there were previous reports regarding sinomenine LC-MS analysis using the positive mode [13,14]. Thus, the positive mode was used for detection. The sinomenine molecular ion *m/z* 330.2 [M+H]^+^ was chosen as parent ion in MRM mode. The MassHunter software “Optimizer” was used to auto-optimize the sinomenine product ion with fragmentor and collision energy. The results showed that *m/z* 181.0 was suitable as the sinomenine product ion at a fragmentor of 180 V and collision energy of 34 V. The transition ion *m/z* 330.2–181.0 was selected for the sinomenine quantitative analysis.

The protein precipitation method was selected for the preparation of the plasma samples because it is easier than solid phase extraction or liquid-liquid extraction, and it can obtain a good recovery. Among acetonitrile precipitation, methanol precipitation, and dichloromethane extraction, the methanol method was selected to remove proteins from the plasma samples based on sinomenine absolute recovery (57.7%, compared to 45.4% and 46.7% in acetonitrile precipitation and dichloromethane extraction, respectively).

Figure 2 shows a typical MRM sinomenine chromatogram in drug-free control plasma, a spiked plasma sample, a RA patient plasma sample collected after transdermal delivery of FTN and a MS2 scan chromatogram of drug-free control plasma. The results show that sinomenine could be found in both the drug-free control plasma sample spiked with sinomenine and in the plasma sample from the RA patient after transdermal delivery of the FTN injection (Figure 2b,c), but cannot be found in the drug-free control plasma (Figure 2a), suggesting that there were no interfering compounds that disturbed the sinomenine detection. The MS2 scan result showed there is no peak found in the range where sinomenine locates, future confirmed this (Figure 2d).

### 2.2. Method Validation

#### 2.2.1. Specificity

As shown in Figure 2, the chromatographic conditions developed were specific for sinomenine determination, showing no interferences from human plasma near the sinomenine retention time. The retention time of sinomenine was 3.17 min.

#### 2.2.2. Linearity and Lower Limit of Quantification

After testing the calibration standards, the equation of calibration curve was calculated as Y = 343.07X + 22.668 (r^2^ = 0.9991), where Y was the peak area of sinomenine and X was the concentration of sinomenine in the sample solution. The linear range is between 0.1173 to 15.02 ng/mL. The human plasma samples from the transdermal delivery study and the QC samples were determined and calculated using the same equation derived from the calibration standards. After the concentrations of sinomenine in the sample solutions were measured, the concentration of sinomenine in human plasma could be calculated. 

The LLOQ was determined to be 0.1173 ng/mL, which is the lowest sinomenine concentration that could be determined with a signal-to-noise ratio of 10.

#### 2.2.3. Precision and Accuracy

The precision and accuracy of the method were determined by analyzing QC samples at three concentrations (0.2347, 1.878, and 15.02 ng/mL) within the standard curve range to validate reproducibility. The coefficient of variance and accuracy were ≤±20% at the lowest concentration and ≤±15% at other concentrations (Table 1). The inter-day CV and accuracy at the LLOQ were 14.2% and 79.7%. These results comply with the requirements in the Guidance of Bioavailability and Bioequivalence Test for Pharmaceutical Preparations, showing that intra- and inter-day precision and accuracy are acceptable [15].

**Table 1:** Intra- and inter-day precision and accuracy of the ultra high performance liquid chromatography coupled with tandem mass spectrometry (UHPLC-MS/MS) method for determining sinomenine concentrations in human plasma (Mean ± SD, *n* = 5).

Condition	Added conc. (ng/mL)	Measured conc. (ng/mL)	Accuracy (%)	Precision (CV, %)
Intra-day	0.2347	0.1880 ± 0.0227	80.1	12.1
	1.878	1.861 ± 0.063	99.1	3.40
	15.02	13.47 ± 0.51	89.7	3.79
Inter-day	0.2347	0.1999 ± 0.0266	85.2	13.3
	1.878	1.901 ± 0.074	101	3.88
	15.02	13.76 ± 0.56	91.6	4.07

#### 2.2.4. Recovery and Matrix Effect

The absolute recovery was determined by comparing the peak areas of the QC samples with the same concentration of sinomenine in the mobile phase. The absolute recovery was calculated as 57.59%–90.82%.

The matrix suppressions for sinomenine at the different concentrations were 6.31%, 1.57% and 4.51%, respectively, which indicated that the matrix suppression had no significant impact on quantification.

#### 2.2.5. Stability

Following a 12 h short-term storage at 4 °C, 30 day long-term storage at −80 °C, and three freeze-thaw cycles, the drug-free control plasma samples spiked with sinomenine at three concentrations were prepared as QC samples and tested. The sinomenine concentrations in the plasma were detected stable (RSD < 15%) (Table 2 and Supplementary Figures).

**Table 2:** The stability of sinomenine in human plasma and solution (Mean ± SD, *n* = 5).

Conditions	Added Conc. (ng/mL)	Measured Conc. (ng/mL)	Precision (CV, %)	Relative Error (%) *
At 4 °C (12 h, plasma)	0.2347	0.2149 ± 0.0219	10.2	−8.43
	1.878	1.965 ± 0.172	8.75	4.63
	15.02	15.02 ± 0.32	2.13	−0.03
At −80 °C (30 days, plasma)	0.2347	0.2181 ± 0.0147	6.75	−7.06
	1.878	1.946 ± 0.148	7.62	3.62
	15.02	15.47 ± 0.46	3.00	2.99
Freeze-thaw cycles (3 times, plasma)	0.2347	0.2389 ± 0.0198	8.27	1.79
	1.878	2.075 ± 0.129	6.22	10.5
	15.02	15.10 ± 0.32	2.15	0.524
At 4 °C (3 days, solution)	0.2347	0.2502 ± 0.0146	5.83	6.60
	1.878	2.112 ± 0.036	1.70	12.5
	15.02	15.36 ± 0.09	0.59	2.24
At 10 °C (24 h, solution)	0.2347	0.2056 ± 0.0119	5.79	−12.4
	1.878	1.931 ± 0.042	2.20	2.83
	15.02	15.41 ± 0.33	2.13	2.60

* The relative error (%) was calculated from the mean value of observed concentration (C_obs_) and nominal concentration (C_nom_) with the following equation: relative error (%) = [(C_obs_ − C_nom_)/C_nom_] × 100%.

Additionally, following 3 days storage in the freezer (4 °C) and 24 h storage in the auto-sampler (10 °C), the sample solution QC samples detected sinomenine concentrations in the acceptable range based on precision with relative error acceptable (<15%) [16] (Table 2 and Supplementary Figures). In general, the results from the stability study indicate that the method was reliable for analyzing sinomenine concentrations in plasma.

### 2.3. Determination of Sinomenine in Human Plasma

The UHPLC-MS/MS method developed in this study was applied to determine the concentration of sinomenine in human plasmas that were collected at varying times before or after FTN transdermal delivery. The results show that sinomenine could be detected in plasma after FTN transdermal delivery in healthy volunteers and RA patients. The concentration of sinomenine in the plasma ranged between 0.4131 and 4.417 ng/mL (Table 3).

**Table 3:** Sinomenine concentration in the plasma samples.

Subject	No.	Concentration * (ng/mL)		
		Before Dosing ^	After Dosing ^	1.5 h after Dosing ^
RA patients	1	- **^†^**	4.407 ± 0.017	-
	2	-	-	1.148 ± 0.074
	3	-	2.245 ± 0.594	-
	4	-	0.6849 ± 0.1007	-
	**Mean**	**-**	**2.446 ± 1.526**	****
Health volunteer	1	-	0.4131 ± 0.0302	-
	2	-	-	-
	3	-	1.514 ± 0.050	-
	4	-	0.5842 ± 0.0000	-
	**Mean**	**-**	**0.8371 ± 0.4837**	**-**

* “concentration” denotes the sinomenine concentration in the plasma, which was calculated based on the sinomenine concentration in the solution multiplied by 2.5 owing to the sample preparation; **^†^** “-” denotes that sinomenine could not be detected in the sample or that the sinomenine concentration in this sample was less than the LLOQ; ^ “before doing” denotes the blood samples were collected before transdermal delivery; “after doing” denotes the blood samples were collected just after 0.5 h of transdermal delivery; “1.5 h after doing” denotes the blood samples were collected at 1.5 h after transdermal delivery.

### 2.4. Discussion

FTN administration via transdermal delivery focused at the lesion sites results in a very low level (ng/mL level) of sinomenine in the plasma in humans. Therefore, a sensitive analytical method was required to quantify sinomenine levels and evaluate its pharmacokinetic properties via transdermal delivery. To achieve this purpose, a sample preparation process that included drying-redissolving process following their precipitation and an UHPLC-MS/MS method were developed to determine the sinomenine concentration in plasma samples. The drying-redissolving process (from 400 μL to 100 μL finally) increased the concentration of the target compound in solution with good recovery in this study. The LLOQ of the developed method was 0.1173 ng/mL in sample solution, which correlates with 0.2933 ng/mL in the plasma, with only 50 μL of plasma being used for sample preparation in this study. Using small amount of sample is the most critical element for pharmacokinetic studies, especially for the study of those subjects with only limited body fluids such as the intra-articular fluids. In this connection, a sensitive, precise and repeatable UHPLC-MS/MS method for the quantification of sinomenine was developed and validated in the current studies. 

While all of the analyses were performed with plasma samples, the “before dosing” samples did not contain any sinomenine, most of the “after dosing” samples contained sinomenine, and only one “1.5 h after doing” sample detected sinomenine. By analyzing the results from the “after dosing” group, the average sinomenine concentration in the “RA” group was higher than that in the “healthy volunteer” group, indicating that there were some differences in joint skin and articular cavity in RA patients compared to the healthy volunteers. By applying this method, we demonstrated that trace amounts of sinomenine can be absorbed into the blood circulation during transdermal delivery of FTN injection. 

## 3. Experimental Section

### 3.1. Materials and Reagents

The reference compound sinomenine was obtained from the National Institute for Food and Drug Control (Beijing, China). The experimental drug FTN injection was provided by the Hunan Zhengqing Pharmaceutical Group Limited (Hunan, China). Acetonitrile (HPLC grade) and methanol (HPLC grade) were purchased from Tedia Company Inc. (TEDIA, Fairfield, OH, USA). Formic acid (MS grade) was purchased from the Merck Group (Merck, Darmstadt, Germany). HPLC grade water was obtained from a MilliQ Ultra Pure Water System (Merck Millipore, Molsheim, France). 

### 3.2. Instrumentation and Analytical Conditions

Analysis was performed on an Agilent 1290 ultra-high performance liquid chromatography system coupled with an Agilent 6460 triple-quadrupole mass spectrometer (UHPLC-MS/MS Agilent Technologies, Santa Clara, CA, USA). For system control, data acquisition, and data processing, the MassHunter software was used. The chromatographic separation was performed on an Acquity BEH Shield C_18_ reverse phase column (1.7 μm, 100 × 2.1 mm I.D.) (Waters, Milford, MA, USA) maintained at 40 °C. Mobile phases were 0.1% formic acid (A) and 0.1% formic acid (B) in acetonitrile with a flow rate of 0.35 mL/min. The gradient elution program was performed using the following procedure: (1) it began with 5% B, increased to 8% over 4 min, and kept increasing to 25% B after 1 min; (2) then it stayed at 25% B for 2 min and then increased to 95% over 1 min and stayed there for 2 min; and (3) then it re-equilibrated to 5% in 0.1 min and stayed there for 2 min. The total method was 12 min with a 10 μL injection volume. 

Mass spectrometry was carried out with an electrospray ionization source (ESI) in the positive ion mode. The following ion source parameters were used: (1) the gas temperature operated at 325 °C with a flow of 11 L/min; (2) nitrogen was used as the nebulizer at 45 psi; and (3) the capillary voltage was +4000 V. The MS recordings were monitored in multiple reaction-monitoring (MRM) mode. After optimization, the fragmenter was 180 V and the collision energy (CE) was 34.0 V for sinomenine. The ions for the MRM analysis of sinomenine were selected at *m/z* 330.2 as the precursor ion and *m/z* 181.0 as the product ion (Figure 1).

### 3.3. Transdermal Delivery Study in Humans

Four healthy volunteers and four patients with rheumatoid arthritis (women with average age of 32.4 ± 8.1 years old) were recruited for the transdermal delivery study at the First Hospital of Hunan University of Chinese Medicine (Hunan, China). Transdermal delivery studies were performed at the proximal interphalangeal joints. The subjects were given a single dose of 50 mg sinomenine hydrochloride (in the form of a FTN injection) for 0.5 h using a transdermal electroporation therapeutic instrument. Before, right after and 1.5 h after the transdermal delivery, blood samples (1.5 mL) were collected into heparinized tubes from a vein and marked as “before dosing,” “after dosing” and “1.5 h after dosing”. The blood was immediately processed to obtain the plasma by horizontal centrifugation at 3000 rpm for 10 min. Then, the plasma was frozen at −80 °C before analysis. The plasma samples before dosing were partly combined and used as drug-free control plasma samples for the method validation. The study protocol was approved by the hospital Ethical Review Committee in accordance with the principles of the Declaration of Helsinki. 

### 3.4. Sample Preparation

The plasma samples were removed from the −80 °C freezer and thawed on ice. Next, 50.0 μL of plasma was diluted to 100.0 μL with water (when preparing calibration and quality control samples, a 25.0 μL working standard solution was added instead of 25.0 μL water). For protein precipitation, 400.0 μL methanol was added to the samples and the samples were vortexed for 30 s and then centrifuged at 12,500 rpm for 10 min at room temperature. A 400.0 μL aliquot of the supernatant was dried in a refrigerated concentrator at 25 °C. The residues were re-dissolved in 100.0 μL of 5% acetonitrile and vortexed for 30 s. After centrifugation at 12,500 rpm for 5 min, 10.0 μL of the supernatant was injected into the UHPLC-MS/MS system for analysis.

### 3.5. Preparation of Standard Solutions and QC Samples

The stock solution of sinomenine was prepared by dissolving the sinomenine reference standard in 0.1% formic acid to a concentration of 19.22 μg/mL. The stock solution of sinomenine was diluted with 0.1% formic acid to produce a serial dilution of working standard solutions containing sinomenine at 0.5865, 1.173, 2.346, 4.692, 9.385, 18.77, 37.54 and 75.08 ng/mL. All of the standard solutions were stored at 4 °C until use.

The plasma standards were prepared by spiking 25.0 μL of each working standard solution into 50.0 μL of drug-free control plasma. These standard solutions were used to construct calibration curves for the quantification of sinomenine in the plasma at a range of concentrations including 0.1173, 0.2347, 0.4694, 0.9388, 1.878, 3.755, 7.510, and 15.02 ng/mL by following the method in Section 3.4. The quality control (QC) samples were prepared in the same manner with sinomenine concentrations of 0.2347, 1.878 and 15.02 ng/mL, respectively. 

### 3.6. Method Validation

#### 3.6.1. Specificity

The drug-free human plasma samples were analyzed to detect potential interferences that could affect the sinomenine analysis due to their retention times and MRM responses.

#### 3.6.2. Linearity and Lower Limit of Quantification (LLOQ)

The sinomenine calibration curve in human plasma was prepared using 8 concentrations, *i.e.*, 0.1173, 0.2347, 0.4694, 0.9388, 1.878, 3.755, 7.510 and 15.02 ng/mL. The linearity of the calibration curve was determined by plotting the peak area (Y) *versus* the prepared analyte concentrations (X). Least squares linear regression analysis was used to determine the calibration curve and the determination coefficient (R^2^). The determination coefficient of the calibration curves should not be less than 0.99. The lower limit of quantification (LLOQ) was defined as the lowest sinomenine concentration that could be determined with a signal-to-noise ratio of 10. The inter-day precision and accuracy of LLOQ were also evaluated with the methods described under 3.6.3.

#### 3.6.3. Precision and Accuracy

The intra-day and inter-day precision and accuracy were determined by analyzing five QC samples at three different concentrations, *i.e.*, 0.2347, 1.878 and 15.02 ng/mL. For the intra-day and inter-day analyses, five replicates of QC samples at three concentrations were detected five times in one day and one time in four different days, respectively. For acceptable intra-day and inter-day values, the accuracy should be between 85% and 115% except for the lower limit of quantification (LLOQ), where the accuracy should be between 80% and 120% [15]. 

#### 3.6.4. Recovery and Matrix Effect 

The absolute recovery of sinomenine from the plasma through the protein precipitation procedure was determined by comparing the peak area from the plasma samples spiked with a known amount of standard in the drug-free plasma with the peak area of the corresponding standard with the same amount of the standard in the mobile phase.

The matrix effect was defined as the ion suppression or enhancement on the ionization of the analytes due to the matrix. To evaluate the matrix effect on the determination of sinomenine, the peak areas of sinomenine in water or in the extracted blank matrix at the same concentration were compared. 

#### 3.6.5. Stability

The stabilities of sinomenine in human plasma and the sample solution were tested using QC samples at three different concentrations under the following four conditions:

Short-term stability—Evaluated by determining the QC samples that were kept at 4 °C for 12 h before sample preparation. The time range covered the maximum span for the plasma preparation.

Long-term stability—Assessed by determining the QC samples that were kept frozen at −80 °C for 10, 20, or 30 days before sample preparation.

Freeze–thaw stability—Measured by analyzing the QC samples that had undergone three freeze (−80 °C, 24 h) and thaw (room temperature) cycles. 

Post-preparative stability—Tested by analyzing the QC samples post-preparation that were kept in the freezer (4 °C) for 0, 3 days or in the auto-sampler (10 °C) for 0, 4, 8, 12, or 24 h.

## 4. Conclusions

In conclusion, an optimum quantitative method has been established, effectively for determining the pharmacokinetic parameters and tissue distribution of sinomenine with trace amount of drug concentrations, like in the articular cavities by using transdermal delivery. Also, such a way of drug delivery would be helpful for designing more ideal regime of FTN for treating RA and other arthritic diseases.

## Supplementary Materials

Supplementary materials can be accessed at: http://www.mdpi.com/1420-3049/20/04/6454/s1.
