# Supplementary material for: An Ultra-High Performance Liquid Chromatographic-Tandem Mass Spectrometric Method for the Determination of Sinomenine in Human Plasma after Transdermal Delivery of the Zhengqing Fengtongning Injection
Source: Molecules. 2015 Apr 10;20(4):6454–65. doi: 10.3390/molecules20046454 (PMC6272659; doi:10.3390/molecules20046454)
Supplement: Supplementary file 1 [file molecules-20-06454-s001.pdf]

Supplemental Materials

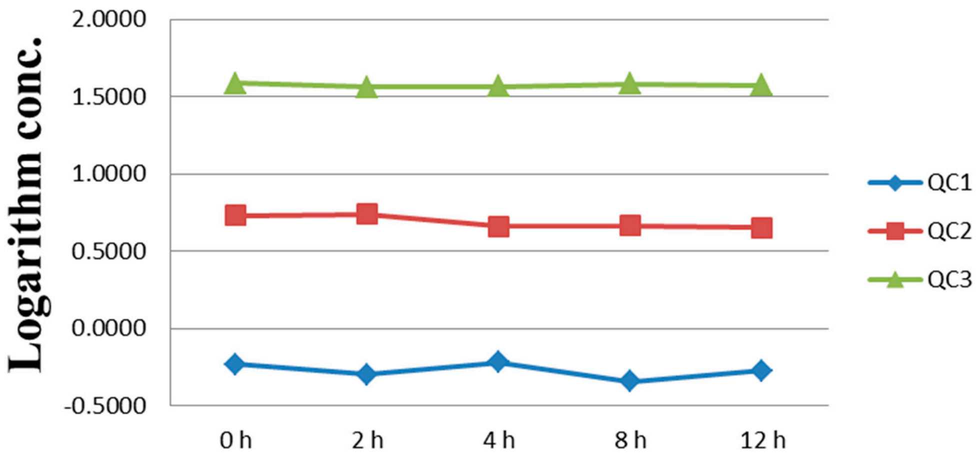

Figure S1. The stability of plasma sample under 4 °C. QC1, QC2 and QC3 means the QC samples with concentration of sinomenine were 0.2347, 1.878, and 15.02 ng/mL, respectively.

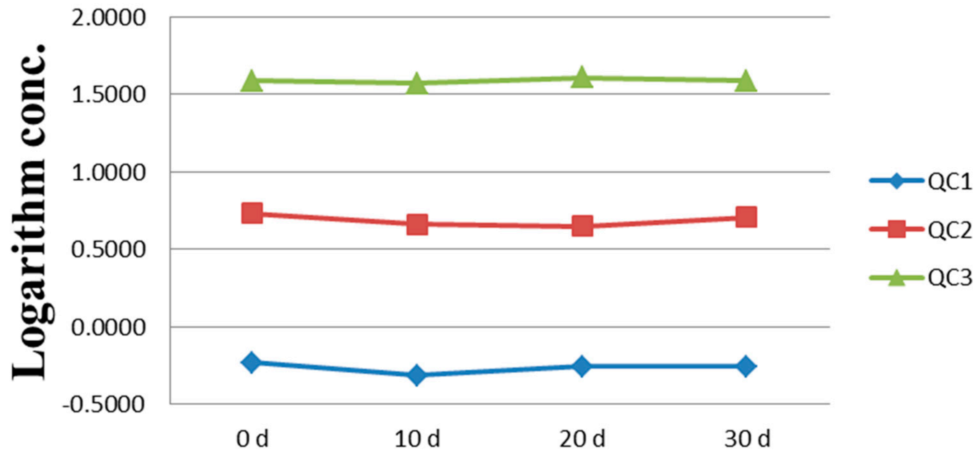

Figure S2. The stability of plasma sample under −80 °C.

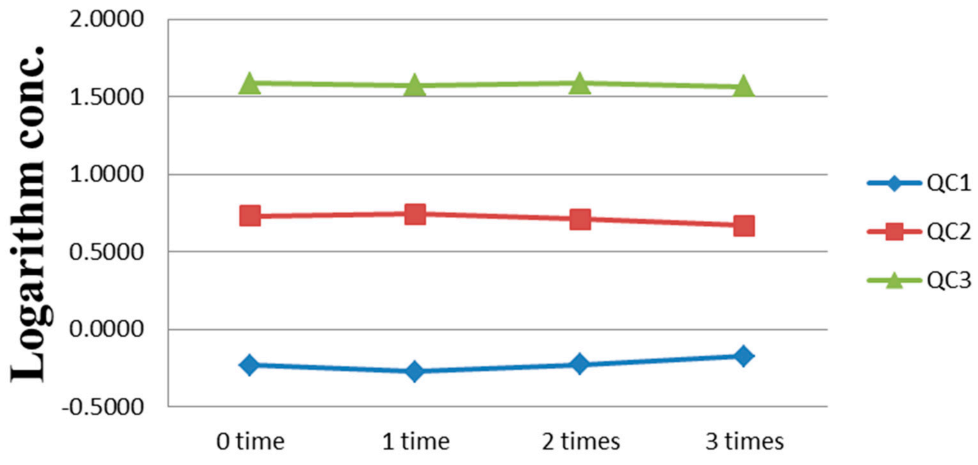

Figure S3. The stability of plasma sample in freeze-thaw three times.

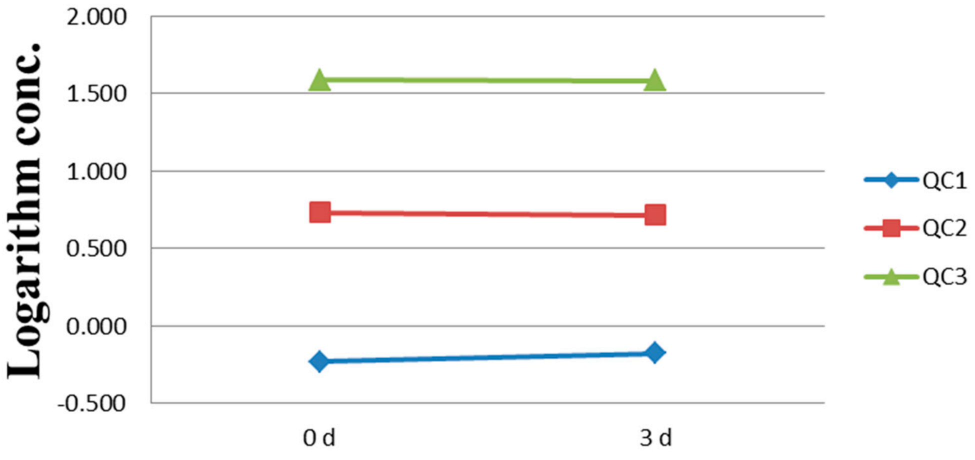

Figure S4. The stability of test sample under 4 °C.

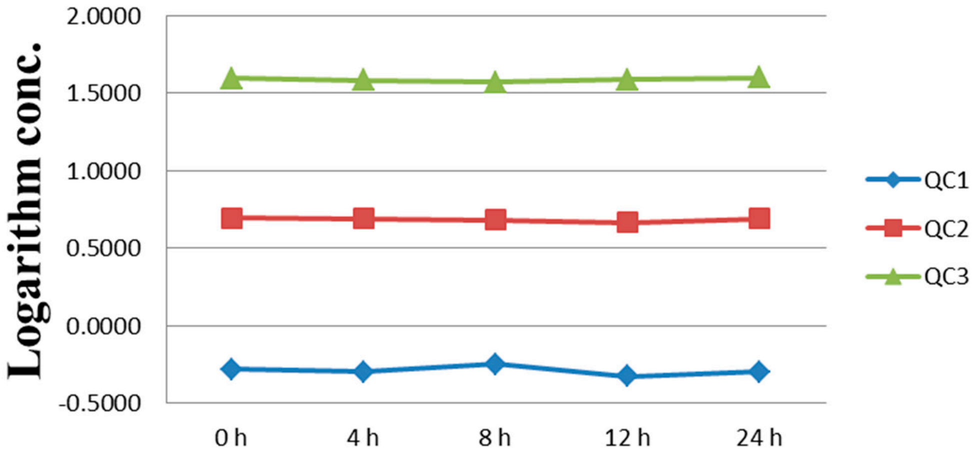

Figure S5. The stability of test sample under 10 °C.
